# Effect of Nigeria Presidential Task Force on COVID-19 Pandemic, Nigeria

**DOI:** 10.3201/eid2813.220254

**Published:** 2022-12

**Authors:** Omotayo Bolu, Boss Mustapha, Chikwe Ihekweazu, Mukthar Muhammad, Assad Hassan, Ahmad Abdulwahab, Adeyelu A. Asekun, Reward Nsirim, Emeka Okechukwu, Ibrahim Attah, Mahesh Swaminathan, Stacie Greby, Adebimpe Adebiyi, Morenike Alex-Okoh, Tochi Okwor, Elsie Ilori, Nwando Mba, Joe Mutah, James Akujobi, Ndirpaya Battah, Wilfred Haggai, Geoffrey Okatubo, Awele Okigbo, Evelyn Castle, Ibrahim Abubakar, Charles Akataobi, Olusegun Adekunle, Sani H. Aliyu

**Affiliations:** Centers for Disease Control and Prevention, Atlanta, Georgia, USA (O. Bolu, A.A. Asekun, M. Swaminathan, S. Greby);; Nigeria Centre for Disease Control, Abuja, Nigeria (B. Mustapha, C. Ihekweazu, T. Okwor, E. Ilori, N. Mba, O. Adekunle);; Office of the Secretary to the Government of the Federation, Abuja (B. Mustapha, O. Adekunle);; Presidential Task Force on COVID-19, Abuja (M. Muhammad, A. Hassan, A. Abdulwahab, I. Attah);; US Agency for International Development, Abuja (R. Nsirim, E. Okechukwu);; Federal Ministry of Health, Abuja (A. Adebiyi, M. Alex-Okoh, G. Okatubo);; Ministry of Information and Culture, Abuja (J. Mutah);; Nigeria National Emergency Management Agency, Abuja (J. Akujobi);; Federal Ministry of Industry Trade and Investment, Abuja (N. Battah);; Federal Ministry of Aviation, Abuja (W. Haggai);; Credo Advisory, Abuja (A. Okigbo);; eHealth Africa, Freetown, Sierra Leone (E. Castle);; Public Health England, London, UK (I. Abubakar);; African Field Epidemiology Network Nigeria, Abuja (C. Akataobi);; Cambridge University Hospitals NHS Foundation Trust, Cambridge, UK (S.H. Aliyu)

**Keywords:** COVID-19, coronavirus disease, SARS-CoV-2, severe acute respiratory syndrome coronavirus 2, viruses, respiratory infections, zoonoses, Nigeria, COVID-19 policy, COVID-19 mitigation, pandemic response

## Abstract

Nigeria had a confirmed case of COVID-19 on February 28, 2020. On March 17, 2020, the Nigerian Government inaugurated the Presidential Task Force (PTF) on COVID-19 to coordinate the country’s multisectoral intergovernmental response. The PTF developed the National COVID-19 Multisectoral Pandemic Response Plan as the blueprint for implementing the response plans. The PTF provided funding, coordination, and governance for the public health response and executed resource mobilization and social welfare support, establishing the framework for containment measures and economic reopening. Despite the challenges of a weak healthcare infrastructure, staff shortages, logistic issues, commodity shortages, currency devaluation, and varying state government cooperation, high-level multisectoral PTF coordination contributed to minimizing the effects of the pandemic through early implementation of mitigation efforts, supported by a strong collaborative partnership with bilateral, multilateral, and private-sector organizations. We describe the lessons learned from the PTF COVID-19 for future multisectoral public health response.

After COVID-19 emerged in China, and before the first case in Nigeria was confirmed, the Nigeria Centre for Disease Control (NCDC) established the multisectoral National Coronavirus Preparedness Group (NCPG) to coordinate the country’s preparedness and response efforts (*1*). The Federal Ministry of Health (FMOH) established the Inter-Ministerial Committee on COVID-19 on January 31, 2020 (*1*).

On February 28, 2020, NCDC confirmed the first COVID-19 case in Nigeria. After that confirmation, the NCPG transitioned to an NCDC-led national multisectoral Emergency Operations Centre (EOC), activated at level 3, the highest level of response in the country intended for public health emergencies, requiring national coordination and use of all available resources (*1*). On March 17, 2020, the country’s president established the Presidential Task Force (PTF) on COVID-19 (*2*) with a mandate to coordinate and oversee Nigeria’s multisectoral intergovernmental efforts to contain the spread and mitigate the impact of COVID-19 in Nigeria. The Secretary to the Government of the Federation chaired PTF, and a national coordinator supervised day-to-day management. The membership included leaders from 13 key ministries, departments, and agencies (MDAs); members were the Honorable Ministers of Health, State for Health, Aviation, Humanitarian Affairs, Disaster Management and Social Development, Education, State for Education, Foreign Affairs, Information and Culture, Interior, and Environment; the directors-general of NCDC and Directorate of State Services; the executive director of the National Primary Health Care Development Agency (NPHCDA); and the World Health Organization (WHO) Country Representative. Cabinet-level membership enabled the PTF to focus on high-level political engagement and decision-making.

The PTF coordinated and developed multisectoral frameworks, established budgets, identified funding sources, developed key policy and enforcement measures, ensured national security throughout the response, coordinated response activities with state governments, and managed negative economic effects of the COVID-19 pandemic in Nigeria. The PTF tenure was originally 6 months; it was extended to December 31, 2020, and then to March 31, 2021. In April 2021, PTF activities were streamlined (by reducing number of MDAs) and transitioned to a Presidential Steering Committee (PSC) on COVID-19, focused on sustaining the multisectoral response as the pandemic waned; this structure freed resources for other health and social issues.

To achieve its mandate, the PTF developed the National COVID-19 Pandemic Multisectoral Response Plan (NPRP) (*3*). Its strategic objectives were to provide a coordinated national and subnational multisectoral response to the COVID-19 pandemic; to reduce COVID-19–related illness and deaths; to mitigate pandemic-related impacts on critical, economic, and health infrastructure; and to support postpandemic recovery and rehabilitation. We describe lessons learned from the Nigeria PTF-guided multisectoral COVID-19 response that may be applicable for future public health responses.

## The National Pandemic Response Center

**Figure 1:**
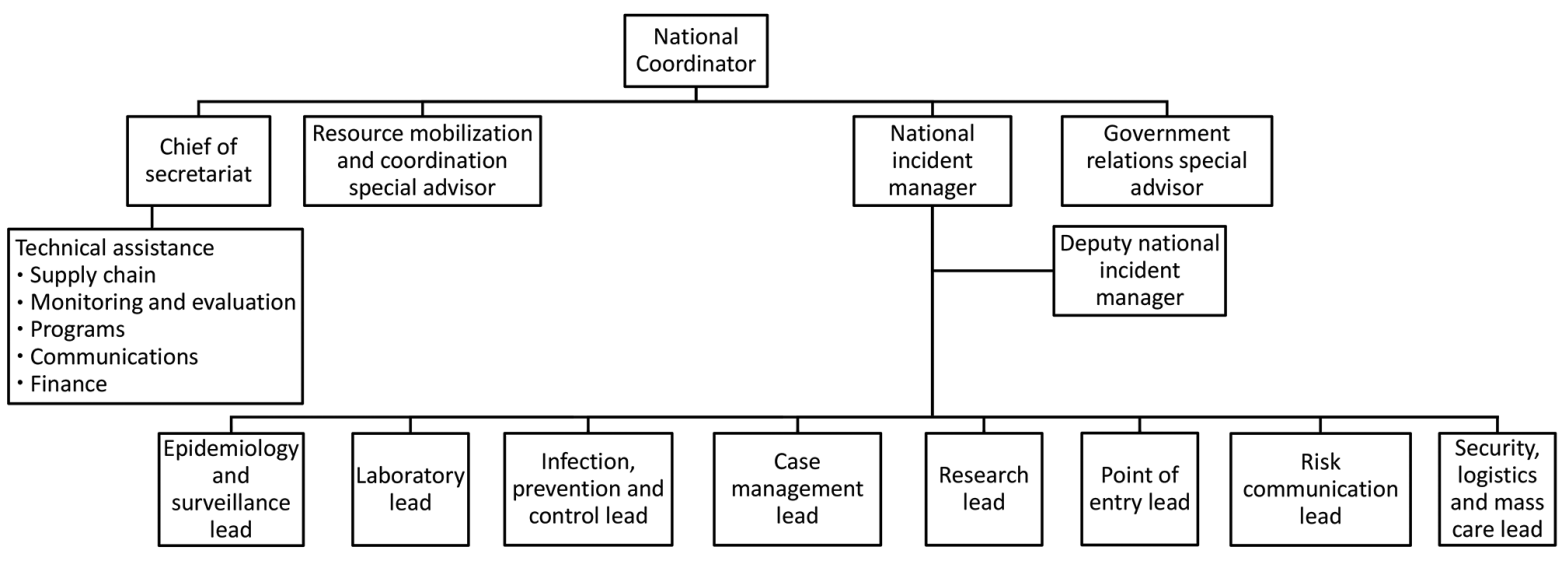
National Pandemic Response Center organizational chart, Nigeria, 2020. Technical assistance comprises staff from various agencies and government who helped oversee each of the specified area listed—supply chain, monitoring and evaluation, programs, communication, and finance.

The PTF established the National Pandemic Response Centre (NPRC), the technical coordinating structure responsible for providing strategic guidance on the national response, estimating MDA resource needs and allocations, and coordinating all response stakeholder efforts. Stakeholders included MDAs, donors, development partners, nongovernment organizations, and civil society. The organized private sector established the Coalition Against COVID-19 (CACOVID) to coordinate their engagement. The NPRC, led by the PTF national coordinator, included Secretariat, led by a chief of secretariat (CoS) and an incident manager, who coordinated 9 functional pillars. Each pillar was led by different government MDAs with mandate and oversight for their pillar; for example, NCDC oversaw surveillance and laboratory and FMOH led case management (Table 1; Figure 1). Staff from the US Centers for Disease Control and Prevention (CDC), US Agency for International Development (USAID), WHO, UNICEF, e-Health Africa, CREDO, and the Bill and Melinda Gates Foundation supported the NPRC.

**Table 1:** National Pandemic Response Center (NPRC) pillars by lead agency and focal area, Nigeria, 2020

Thematic area	Lead agency	Area of focus
Epidemiology and surveillance	Nigeria Centre for Disease Control	Improve surveillance, early detection and timely reporting of community transmission of COVID-19
Laboratory	Nigeria Centre for Disease Control	Strengthen laboratory testing and performance
Point of entry	Federal Ministry of Health; Port Health Services	Expand border security patrol and ensure every entry point are equipped for sample collection for testing
Case management	Federal Ministry of Health	Provide technical assistance and epidemiological support to states to improve case management
Infection, prevention, and control	Nigeria Centre for Disease Control	Embed and strengthen functional infection prevention and control programs across the country
Risk communication and community engagement	Federal Ministry of Information and Culture	Strengthen communication around COVID-19 and continuously work with partners to undertake research to address key drivers of behavior change
Security, logistics, and mass care	Federal Ministry of Humanitarian Affairs, Disaster Management, and Social Development	Develop standards and criteria for enforcement of protocols and sanctions
State coordination and government relations	Nigeria Governor’s Forum	Strengthen state engagement and ownership of the COVID-19 response efforts
Resource mobilization	Office of the Secretary of the Federation	Ensure sustainable funding for the COVID-19 response
Research	Federal Ministry of Health	Conduct scientific, clinical, anthropological and socio-economic research to provide the evidence base for guiding decision making in COVID-19 planning and response in Nigeria
Sustainable production subgroup	Federal Ministry of Industry, Trade and Investment	Ensure long-term uptake and availability of COVID-19 supplies and products from Government and credible investors

The PTF convened a multidisciplinary advisory group to provide evidence-based briefing papers, informing real-time decision making. The group comprised health policy and service experts, including epidemiologists, modelers, public health experts, social scientists, foreign and domestic academicians, and NPRC staff. This group produced >44 papers supporting PTF decision making by modeling the epidemic trajectory, including potential number of expected cases, deaths, and bed space, and recommended policies and interventions required to reduce transmission risk (*4*).

### The NPRC

To provide effective technical guidance and direction, the NPRC developed a comprehensive pandemic response plan (PRP), the blueprint for the coordinated national COVID-19 strategy (*3*), in addition to the NCDC-developed public health incident action plan. The PRP included activities beyond health, such as disaster management, humanitarian affairs, information, security, finance, trade, and investment. The PRP described complementary response roles of national and state governments (Table 2), private sector, and development partners. The PRP divided the response into 6 phases based on the national and WHO epidemic response plans (Table 3), with specific tasks for each phase following specific trigger events.

**Table 2:** The COVID-19 multisectoral ministries, departments, and agencies by sector and role, Nigeria, 2020

Sector	*National MDAs and roles*	*State MDAs and roles*
Health	NCDC: Lead on epidemiology, surveillance laboratory, and IPC	The state Emergency Operations Center, which comprises members from the State Ministry of Health, State Primary Healthcare Development Agency, and State Health Management Board liaise with the FMOH and NCDC in their day-to-day operations
	FMOH: Lead on case management port health management, research on COVID-19	
	Nigeria Primary Healthcare Development Agency: Community centers and vaccination	
	Nigeria Institute of Medical Research: Research around COVID-19	
Nonhealth	Ministry of Information and its agencies take the lead on risk communication	The state task force, led by the governor or his designate, includes representatives from line MDAs similar to those in the national MDAs—these include the ministries of information and finance, the State Emergency Management Authority, the security agencies, and others
	Ministries of Interior, Aviation, and Transportation and their agencies, such as Nigeria Immigration Service, Customs, Nigeria Civil Aviation Authority, Federal Airports Authority of Nigeria, Nigerian Ports Authority, Nigerian Maritime Administration and Safety Agency, run the ports of entry	
	Ministry of Humanitarian Affairs and its agency, National Emergency Management Authority, lead mass care	
	Ministry of Defense and the Police, Nigeria Security and Civil Defence Corp, and Federal Road Safety Corp support security and logistics	
	Ministry of Industry, Trade, and Investment oversee and coordinate local production of personal protective equipment	
	Ministry of Finance is involved in economic impact and recovery activities	

*FMOH, Federal Ministry of Health; MDA, ministries, departments, and agencies; NCDC, Nigeria Centre for Disease Control.

**Table 3:** Summary of the national pandemic response plan phases, Nigeria, 2020

Phase	Description	Response
1	No cases	Monitoring global trends, surveillance, early detection of high-risk passengers for follow-up, isolation of symptomatic cases
2	Sporadic cases	Increased surveillance, set up of quarantine and isolation procedures, cancellation of gatherings, public sensitization, forecasting and quantification of commodities and personal protective equipment
3	Cluster of connected cases	Intensified surveillance towards containment, expedited sample collection, testing; isolation and management of suspected/confirmed cases, mass care
4	Community transmission	Declaration of a national emergency activation of triage sites and alternative treatment sites. Increased stock of supplies. Mass care. Allocation of resources for public safety and order
5	Postpeak	Continued surveillance, testing, case management, and infection prevention and control measures. Provision of social protection services
6	Recovery	Deactivation and decontamination or triage and treatment sites. Reviewed and modified risk communication. Authorization for opening of public spaces

## The PTF Midterm Review

In July 2020, four months after it began, the NPRC implemented a midterm review (MTR) to assess PTF achievements and challenges and adapt the response for the remaining PTF term (*5*). To complement ongoing public telephone and online surveys and data from each pillar, we conducted an online survey with key stakeholders from MDAs, multilateral organizations, donors, and civil society to assess and score perceptions on the PTF’s performance. The survey revealed an aggregate 4.0/5.0 score for the PTF’s role in coordinating the national COVID-19 response and collaboration with stakeholders. The lowest score was 2.5 for mitigating the socioeconomic impact (*5*).

During the MTR, MDAs and pillars presented performance reports and received feedback from stakeholders including cabinet ministers, legislators (including the chairmen of the relevant committees on health in the Senate and House of Representatives), representatives from the Office of the Vice President, the Nigeria Governors’ Forum (NGF), donors and partners, the diplomatic community, civil society, and Health Sector Union representatives.

The MTR findings (*5*) revealed PTF achievements, with collaboration of NCDC and FMOH, that included the setup of 39 laboratories nationwide and of 131 treatment centers with 7,040 total bed capacity and 256 ICU beds. A total of 138,462 persons were tested and treated, representing a 40% increase in testing from March to June 2020 (*5*). Challenges identified in the MTR included delays in international border closures, negative socioeconomic effects including access to essential services and resources for livelihood, insufficient contact tracing, and low testing rates (<1% of the population had tested by the time of the MTR) (*5*). MTR findings were used to update the NPRP, guiding the national COVID-19 response through detailed action plans for implementation by pillars over the subsequent 6 months.

## The PTF End-of-Year Review

In December 2020, the PTF conducted an end-of-year review to assess performance, identify how to sustain the gains made to date, and plan for PSC initiation. An online survey was distributed to key stakeholders to gather feedback on the government of Nigeria’s pandemic response. The survey revealed that stakeholders were satisfied with the PTF’s performance and commitment to the COVID-19 response. By the end-of-year review, key achievements included developing the Nigeria International Travel Portal (NITP); ramp-up of testing; enhanced infection, prevention, and control (IPC) practices for healthcare workers; reduced infections in healthcare workers; sufficient bed space for case management;, and resumption of economic activities. Challenges included poor enforcement of nonpharmaceutical preventive measures, low community-level testing, and slow economic recovery (*6*).

### End-of-Year Review Findings

**Figure 2:**
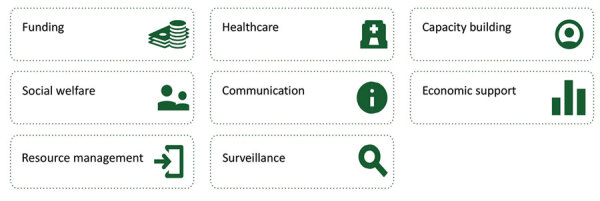
Depiction of the Presidential Task Force on COVID-19 end-of-year review categories, Nigeria, 2020.

During March–December 2020, the PTF, through different pillars and the Secretariat, continued fund mobilization, policy formulation, and public- and private-sector collaboration to improve laboratory testing capacity, preventive measure enforcement, healthcare infrastructure improvement, and capacity development. We organized PTF achievements into 8 categories (Figure 2).

#### Funding

**Figure 3:**
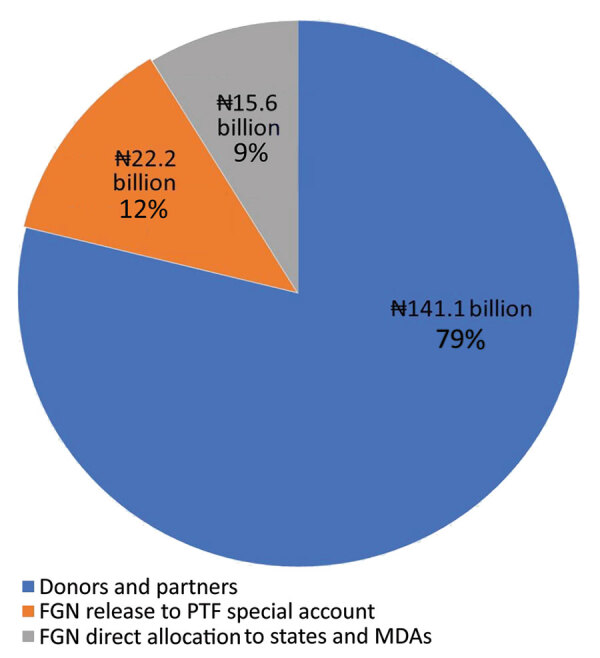
Percentage contribution in naira and source of funds for COVID-19 response, Nigeria, March–December 2020. FGN, federal government of Nigeria; PTF, Presidential Task Force; MDAs, ministries, departments, and agencies. Source: United Nations Development Program dashboard, Office of the Secretary to the Government of the Federation, Resource Mobilization pillar.

A total of ₦178,800,260,723 (458,462,207 USD) was mobilized for the COVID-19 response (Figure 3). Local and international donors/partners contributed >70% of funds. To promote fiduciary transparency, donors were encouraged to provide direct support to state-led activities based on state plans to avoid funds passing through PTF or national government accounts. This support did not include funds spent directly by donors, e.g., building isolation centers, hiring staff, and deploying rapid-response teams.

#### Healthcare

By December 31, 2020, the PTF, through NCDC and other partners, had successfully supported 975,786 COVID-19 tests, a 600% increase from the 138,462 tests recorded at the MTR in June 2020. PTF reported 13,798 active cases and 1,311 deaths for a case fatality rate of ≈2% (*7*). Daily COVID-19 trends were reported, and weekly epidemiologic profiling guided allocation of resources.

#### Facility and Laboratory Capacity Building

At the beginning of the pandemic, frontline HCWs were trained and provided with infrastructure for managing suspected cases while adhering to safety protocols and procedures. Capacity development focused on 3 categories: isolation and treatment facilities, COVID-19 testing capacity, and training. The PTF, in collaboration with NCDC, private-sector partners (especially CACOVID), and some state governments, successfully set up and accredited 131 isolation and treatment centers with a total capacity of 7,040 beds across the country over a period of 6 months.

The NCDC led development of decentralized COVID-19 testing laboratory capacity to ensure nationwide geographic coverage and improve 24-hour test turnaround time. By December 31, 2020, Nigeria had 98 operational laboratories, 68 government-owned and 30 private/corporate owned, a 151% increase since the MTR. Molecular testing platforms varied, some, such as TB GeneXpert (Cepheid, https://www.cepheid.com), were repurposed for COVID-19 testing; genomic sequencing was instituted in 3 laboratories. Through December 5, 2020, more than 35,500 HCWs were trained on appropriate IPC practices, including proper use of personal protective equipment. FMOH led case management and developed protocols for establishment of isolation and treatment centers with federal tertiary hospitals as training hubs. Training protocols for frontline HCWs on treatment guidelines and capacity building of biomedical engineers on ventilators and other devices were established. Regular national case reviews of state situations became standard.

#### Social Welfare

To ensure support for those in need, a national economic recovery plan was created in June 2020 (*8*). The government developed programs to aid vulnerable persons and households. Donors/partners (e.g., CACOVID), MDAs (e.g., Federal Ministry of Humanitarian Affairs, Disaster Management, and Social Development), supported provision and distribution of palliative packages to those in need, including low-income internally displaced and physically challenged persons and women, children, and elderly, that were intended to cushion socioeconomic and psychological effects of the COVID-19 pandemic. Palliatives were either food items delivered to frontline states (a total of 70,000 megatons distributed to 8,827,129 households in 24 states) or cash transfers (1,289,405 beneficiaries in 34 states and the Federal Capital Territory [FCT]).

The distribution of palliatives coincided with a period of social unrest and protests against police brutality (the #EndSARS [Special Anti-Robbery Squad] movement). The harsh economic environment due to the pandemic and public mistrust of government intentions further exacerbated the unsettled mood in the country. Widespread looting of relief items from storage sites and warehouses followed. State governments were widely criticized for not distributing the relief items earlier; however, CACOVID had not completed the delivery of some palliatives and had to halt the process when protests started. Furthermore, the size of the country and level of poverty meant that relief items were insufficient to reach all in need.

#### Communication

The risk communication pillar maintained regular communication with stakeholders, engaging partners and donors, MDAs, and the public. The PTF leveraged traditional and social media for communication and advocacy materials to build awareness of the COVID–19 pandemic and Nigeria’s response. Communication was highlighted as a success of the PTF in >24 rounds of nationwide and state weekly telephone polls and surveys conducted by NOI Polls during May–November 2020, assessing citizens’ perception of national and state government’s COVID-19 response. State-specific sample sizes ensured states were proportionately represented in surveys of >36,000 citizens. The surveys assessed citizens’ perception in 5 key areas: trust/concern, communication monitoring, preventive measures/palliatives, misinformation, and testing. Topics covered included adherence to COVID-19 prevention protocols (e.g., mask-wearing, handwashing, physical distancing); burial protocol, school and National Youth Service Corps (NYSC) reopening, and exposure to and effect of COVID-19 messages. Data collected revealed that PTF successfully maintained regular communication with relevant stakeholders and the public. Challenges included delayed release of guidelines, government mistrust, noncompliance with COVID-19 protocols, and poor testing uptake. As vaccines came into sight, PTF worked with NPHCDA to update the communication strategy to address vaccine uptake and hesitancy.

## Economic Support

 To cushion the effect of lockdowns and restrictions in movement, the federal government’s economic program provided loans for individual persons and small and medium-size businesses (Table 4). In addition, at the beginning of the pandemic, travel and tourism, education, worship centers, restaurants, and other sectors were closed at different periods to minimize the risk for transmission and safeguard the health of citizens. Stay-at-home orders and cessation of nonessential movements and activities were initially mandated for Lagos, Ogun, and FCT. Subsequently, other states adopted varying degrees of lockdown strategies, including school closure, movement restriction, and cessation of interstate and international travel (*6*). As lockdowns eased and businesses, schools, and other places for social gatherings began to operate, guidelines for the safe and efficient reopening of the Nigeria economy were developed and released in tandem with the Economic Sustainability Plan (*8*).

**Table 4:** COVID-19 economic support, Nigeria, March–December 2020

Government Enterprise and Empowerment Program (GEEP)	Trader and market Moni loans	Rapid expansion of the National Social Register
Sensitized over 5 million small scale traders listed in the GEEP database about COVID-19 pandemic. Granted 3 months moratorium on payments owed to 2.2 million existing GEEP beneficiaries and completed 99% of the loan disbursement target	Disbursed loans to 43,117 beneficiaries in 11 states (Lagos, Ogun, Plateau, Bauchi, Yobe, Zamfara, Katsina, Edo, Cross River, Enugu, and Imo) and the Federal Capital Territory	Updated the National Social Register to include previously unidentified vulnerable citizens, now including 3.6 million households in 36 states and the Federal Capital Territory

The PTF worked closely with the Federal Airports Authority, Nigeria Immigration Service, the Nigerian Civil Aviation Authority, and other international aviation partners and authorities to develop best-practice protocols and measures for resuming air travel. The NITP was updated with changes in travel policies based on the epidemiology in Nigeria and other countries.

NYSC, a national service for new university and college graduates, reopened orientation camps on May 17, 2020. To ensure the safety of the staff and volunteers, PTF, NCDC, and NYSC teams assessed COVID-19 control measures before opening the camps that could host >1,000 participants. Public health guidance for safe reopening of the NYSC camps was distributed across the country. With support from NCDC, all participants underwent COVID-19 PCR or rapid antigen testing before or upon arrival at the camp. Trained staff were assigned to NYSC camps to monitor and support compliance with COVID-19 preventive measures. NYSC camp residents were placed in bubbles, with minimal outside contact after COVID-19 screening to reduce risk for COVID-19 transmission; persons testing positive were not admitted.

After the PTF announced reopening of places of religious and social gatherings, NCDC published guidelines to reduce the risk for infection in places of worship, social gathering centers, hotels, and event centers. Guidelines included mandatory use of face masks, temperature screening, denial of entry for sick persons, provision of hand hygiene stations, attendance limited to a third of the seating capacity, physical distancing of >2 m, and no physical contact (*9*). Enforcement proved to be challenging and depended on state governments to implement and sanction organizations and entities that did not comply with the guidance.

## Surveillance

The PTF surveillance and epidemiology pillar, led by NCDC, intensified surveillance activities for early COVID-19 case detection, timely reporting of cases, and coordination of the outbreak response. The pillar provided daily epidemiologic updates, weekly profiling, trend analysis, and summaries to aid the PTF in informed decision making. The response was conducted in 3 phases: prevention and preparedness, containment, and control and mitigation.

### Prevention and Preparedness

 The NCDC’s surveillance and epidemiology pillar trained and deployed staff to support preparedness for all states. Working with FMOH Port Health Services leadership, they identified point-of-entry sites, developed guidelines and data collection tools, and put the tools into use. They designated treatment centers and conducted simulation exercises with multiple stakeholders.

### Containment

The national preincident action plan NCDC developed and referenced in the NPRP was adapted by states. Rapid response teams deployed by NCDC supported affected states with screenings at points of entry and contact tracing activities in high-priority states. States followed travel restrictions and lockdown protocols for nonessential activities.

### Control and Mitigation

The pillar revised the national COVID-19 case definition and enabled community active-case search, which increased identification of COVID19 infected individuals based on symptoms and testing. COVID-19 treatment centers were strengthened to ensure mandatory institutional quarantine and testing for international travelers was enforced. In addition, policies on home-based care for COVID-19 drastically reduced bed occupancy in isolation centers and increased the capacity of healthcare workers to manage patients who needed emergency care. However, when self-isolation and IPC were not strictly adhered to, home-based care increased the risk for transmission of COVID-19 among family members and the community (*10*). Hotspots were identified across local government areas, and efforts were intensified to contain community transmission.

### Resource Management

Funds and resources were received from public and private-sector organizations including national and state governments, CACOVID, WHO, UNICEF, the US government (CDC, USAID, US Department of State, Walter Reed Army Institute of Research WRAIR), the UK Department for International Development, the European Union, the Government of Japan, and other partners (*5*). Mobilized donor funds came from the One UN COVID-19 Response Basket Fund. Resources included technical support and expertise to ensure comprehensive COVID-19 response. Areas supported were technical guidance on IPC measures, training healthcare workers on case management and outbreak response, risk communication to mitigate disease spread, community engagement and awareness of COVID-19 prevention measures, civil society organization mobilization to sustain essential health services, strengthened state level surveillance operational capacity, data analysis, and logistics. By December 6, 2020, the partner agencies allocated ₦19,500,000,000 (US $50,000,000) for technical support. Partners donated materials to MDAs and state governments, including medical equipment, consumables, and infrastructure. Although the PTF worked closely with donors to prioritize and implement projects, no donor funds were directly disbursed or spent by the PTF. All data on mobilized resources were shared on the PTF dashboard for accountability and public access (*5*).

### Coordination and Partnership at National and Subnational Levels

The PTF’s collaboration with the donors, partners, and the private sector, especially CACOVID, enabled resources and technical support, such as setting up the NITP and isolation centers at the state level, to be provided in a timely manner. In addition, the National Assembly played a key role in passing legislation for a national COVID-19 budget, and the NGF, made up of governors of all 36 states and FCT, served as a crucial platform for the PTF and Secretariat to get standardized messages and protocols to all for prompt implementation at the subnational level.

At the end of the PTF term in March 2021, the roles and responsibilities were transitioned to the PSC until the end of 2021, mandated to build on the PTF achievements and work with NPHCDA to ensure the successful rollout of COVID-19 vaccines across Nigeria. Despite the success recorded over the period of operation, the PTF had several challenges. Nigeria started the pandemic with a weak healthcare infrastructure, including insufficient intensive care units, isolation centers, and other critical needs to provide care for a disease with potential high hospitalization rates. For example, the country had 293 ventilators, far less than the projected estimated need of 1,769. However, level of hospitalization was significantly less than anticipated; hence, hospitals did not run out of ventilators. Logistic bottlenecks, compounded by COVID-19 mitigation lockdowns and shortages of PPE and COVID-19 test kits or components, were common during the early weeks of the pandemic, decreasing COVID-19 testing capacity when it was critical to increase testing. The PTF formed partnerships with key stakeholders and donors to mitigate these challenges. COVID-19 stigma led to difficulty testing suspected persons and eliciting contacts. Effective risk communication and stakeholder engagements were required to address this challenge. A key lesson learned was the importance of mobilizing and responding quickly to the pandemic as well as reinforcing collaborative work with MDAs, including NCDC and other federal ministries, state governments, and donors/partners. The mode of distribution of palliatives provided by the private sector was challenging because of delays in distribution and #EndSARS protests, resulting in palliatives not reaching all in need. Using a health and demographic surveillance system (HDSS) tool could have addressed some gaps in palliative distribution (*11*).

In addition, better monitoring of travelers and enforcement of nonpharmaceutical interventions, such as mask-wearing, could have improved COVID-19 mitigation efforts. Although the NGF served as a platform to interact with state governments, occasional differences in opinion created challenges for standardization of recommendations. Finally, the Nigeria COVID-19 response was dynamic as knowledge continued to emerge about the disease. However, the strength of the coordination enabled rapid mobilization and deployment of resources nationwide to address the emergency.

## Conclusion

The PTF provided oversight for the multisectoral Nigeria COVID-19 response. Through pillars and functional working groups, the PTF supported coordination and policy formulation, resource mobilization from donors and the private sector, establishment of COVID-19 infrastructures and services, effective risk communication, capacity building of health workers, and improved humanitarian and social interventions. Through the coordinating platform and the development and implementation of policy documents, the PTF contributed to limiting the spread of the virus and mitigating its impact on the health of Nigerians and on the country’s economy. In mid-2021, Nigeria scored 4th of 50 countries on an independent normalcy index reviewing transportation and travel, recreation and entertainment, and retail and work (*12*). To ensure gains were not lost and to continue to have a functioning multisectoral body, the PSC on COVID-19 continued to work closely with key health agencies, including the NCDC and NPHCDA, providing strategic direction and oversight to COVID-19 response efforts. This arrangement not only served the country relatively well amid an extraordinary public health crisis but also strengthened government public health agencies to respond better to future pandemics.
